# Organic Synthesis Mediated by Carbon Nitride Photocatalysis and Photocharged Carbon Nitrides

**DOI:** 10.1021/acs.accounts.5c00687

**Published:** 2025-12-24

**Authors:** Chong Wang, Jingru Zhuang, Oleksandr Savateev

**Affiliations:** Department of Chemistry, 26451 The Chinese University of Hong Kong, Sha Tin, New Territories, Hong Kong, China

## Abstract

Organic synthesis mediated by graphitic carbon nitrides (g-CNs) became a research hotspot primarily due to a combination of the following aspects: (1) the simple and convenient preparation of the material on a gram scale from inexpensive precursors, (2) the heterogeneous nature of the material, which allows for its easy recovery from the reaction mixture, chemical and thermal stability, and possibility to create nanostructures, such as membranes and thin films, and (3) the effective utilization of sustainable energy—photons in the UVA–vis range, which may be used to drive chemical reactions that are endergonic in the dark. By combining various spectroscopic techniques and the results of theoretical modeling, our group identified three modes of substrate activation by g-CN via (1) photoinduced electron transfer, (2) energy transfer, and (3) proton-coupled electron transfer/hydrogen atom transfer. In this Account, we discuss the chemical structure of the electronically excited state of g-CN. This information may be used to design rational pathways for substrate activation via the above-mentioned mechanisms. Using elemental sulfur (S_8_) as a nearly 100% atom-efficient sulfurating agent, we developed a set of methods to incorporate sulfur atom(s) into the organic scaffold by means of g-CN photocatalysis. On the other hand, we identified S_8_ as a more selective, compared to O_2_, sacrificial oxidant to mediate a few net-oxidative photocatalytic transformations. Among the products of the developed synthetic methods are highly fluorescent heterocycles, artificial flavoring agents, and precursors for organic synthesis. While g-CNs are typically used by the community as photocatalysts under continuous light illumination (the sensitizer is regenerated many times in the catalytic cycle), our group contributed to understanding the ability of this class of materials to undergo photocharging, i.e., to store charges by forming long-lived radical species. We applied photocharged carbon nitrides as donors of electrons and protons in the dark in a series of organic transformations. We outline the current challenges and future development prospects of carbon nitride-mediated organic synthesis. At the same time, we provide guidance on the development of organic catalytic systems and material design at the molecular level.

## Graphical abstract

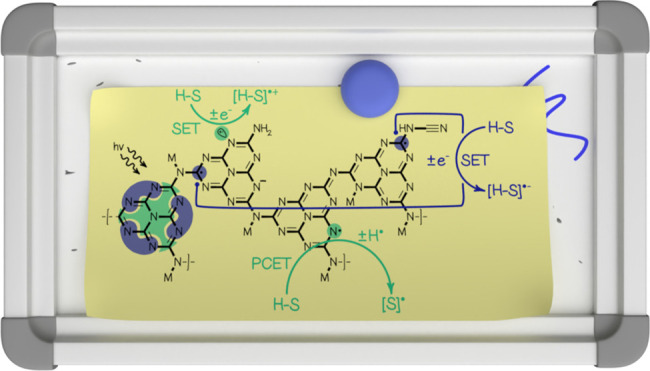

## Key References

-

Savateev, A. ; Tarakina, N. V. ; Strauss, V. ; Hussain, T. ; ten Brummelhuis, K. ; Sánchez Vadillo, J. M. ; Markushyna, Y. ; Mazzanti, S. ; Tyutyunnik, A. P. ; Walczak, R. Potassium Poly­(Heptazine Imide): Transition Metal-Free Solid-State Triplet Sensitizer in Cascade Energy Transfer and [3 + 2]-cycloadditions. Angew. Chem. Int. Ed. 2020, 59, 15061–15068 10.1002/anie.202004747PMC749690432412175.^1^ Fluorescence of ^1^O_2_ in nIR unambiguously proves the generation of this species by potassium poly­(heptazine imide) and hints at singlet–triplet intersystem crossing in this material. ^1^O_2_ mediates the generation of nitrile oxides from oximes and their subsequent [3 + 2] cycloaddition to nitriles.
-

Galushchinskiy, A. ; Zou, Y. ; Odutola, J. ; Nikačević, P. ; Shi, J.-W. ; Tkachenko, N. ; López, N. ; Farràs, P. ; Savateev, O. Insights into the Role of Graphitic Carbon Nitride as a Photobase in Proton-Coupled Electron Transfer in (sp^3^)­C–H Oxygenation of Oxazolidinones. Angew. Chem. Int. Ed. 2023, 62, e202301815 10.1002/anie.20230181536852584.^2^ Carbon nitride is used as an organic photocatalyst, which converts light energy into a driving force for the abstraction of hydrogen atom in oxazolidinones and their conversion into the corresponding 2,4-diones.
-

Savateev, O. ; Nolkemper, K. ; Kühne, T. D. ; Shvalagin, V. ; Markushyna, Y. ; Antonietti, M. Extent of carbon nitride photocharging controls energetics of hydrogen transfer in photochemical cascade processes. Nat. Commun. 2023, 14, 7684 38001091 10.1038/s41467-023-43328-6PMC10674013.^3^ The density of electrons stored in carbon nitrides and the type of counterion affect its reactivity. Photocharged poly­(heptazine imide) featuring ammonium counterions can survive in air for at least half an hour.
-

Wang, C. ; Lin, S. ; Lu, Y. ; Hou, Y. ; Savateev, O. ; Cheng, J. Enhancing Photocatalytic Redox Activity of Polymeric Carbon Nitride for Valuable Fluorinated Heterocycles through Fast-Track Electron Highways. ACS Catal. 2024, 14, 11308–11317 .^4^ Potassium poly­(heptazine imide) is used as a photocatalyst to construct pharmaceutically relevant fluorinated heterocycles. X-ray photoelectron spectroscopy and theoretical calculations confirm the role of potassium ions as catalytic sites for substrate adsorption and enhanced electron transfer.

## Introduction

We consider it essential to highlight several milestone studies on carbon nitrides and photocatalysis, demonstrating their connection with our own research on carbon nitride organic photocatalysis. Like many nanomaterials, a substance or a family of substances that nowadays is referred to as “graphitic carbon nitride” (g-CN) was presumably synthesized for the first time around 200 years ago by Wöhler (Figure 1).^5^ It was not until the beginning of the 21st century that Lotsch and Schnick revealed the crystal structure of this material by employing transmission electron microscopy.^6^ Still, single-crystal X-ray analysis remains challenging due to the crystallite size being limited to a few hundred nanometers. Photocatalysis or photoredox catalysis is a popular area of research nowadays. However, chemists were exercising photochemistry in organic synthesis starting from the 19th century, at least.^7^ The term “photocatalysis” is encountered for the first time in a research article from 1911, authored by Bruner and Kozak.^8^ In the 20th century, interest in graphitic carbon nitrides was revived several times.^9^ Its first application as a photocatalyst, without doubt, is linked to the generation of hydrogen and water splitting, reported by Wang and Antonietti in 2009.^10^ The natural extension of graphitic carbon nitride application in organic photocatalysis was the selective oxidation of alcohols to carbonyl compounds reported by Wang in 2010,^11^ which still remains one of the most popular model reactions to assess the performance of newly synthesized g-CNs. Our research group was established in 2017 at the Max Planck Institute of Colloids and Interfaces, guided by the curiosity to investigate this 200-year-old material as a photocatalyst in organic synthesis, on the one hand, and the need to create more sustainable methods in organic synthesis on the other. Until now, the group’s research output may be divided into a few topics. A few of them, such as the application of elemental sulfur and photocharged carbon nitrides in organic synthesis, are highlighted in this Account. Upon relocation to the Chinese University of Hong Kong in 2023, the group continues research in carbon nitride organic photocatalysis while at the same time introducing and adopting innovative approaches in chemical education, such as educational video gaming.^12^

**Figure 1:**
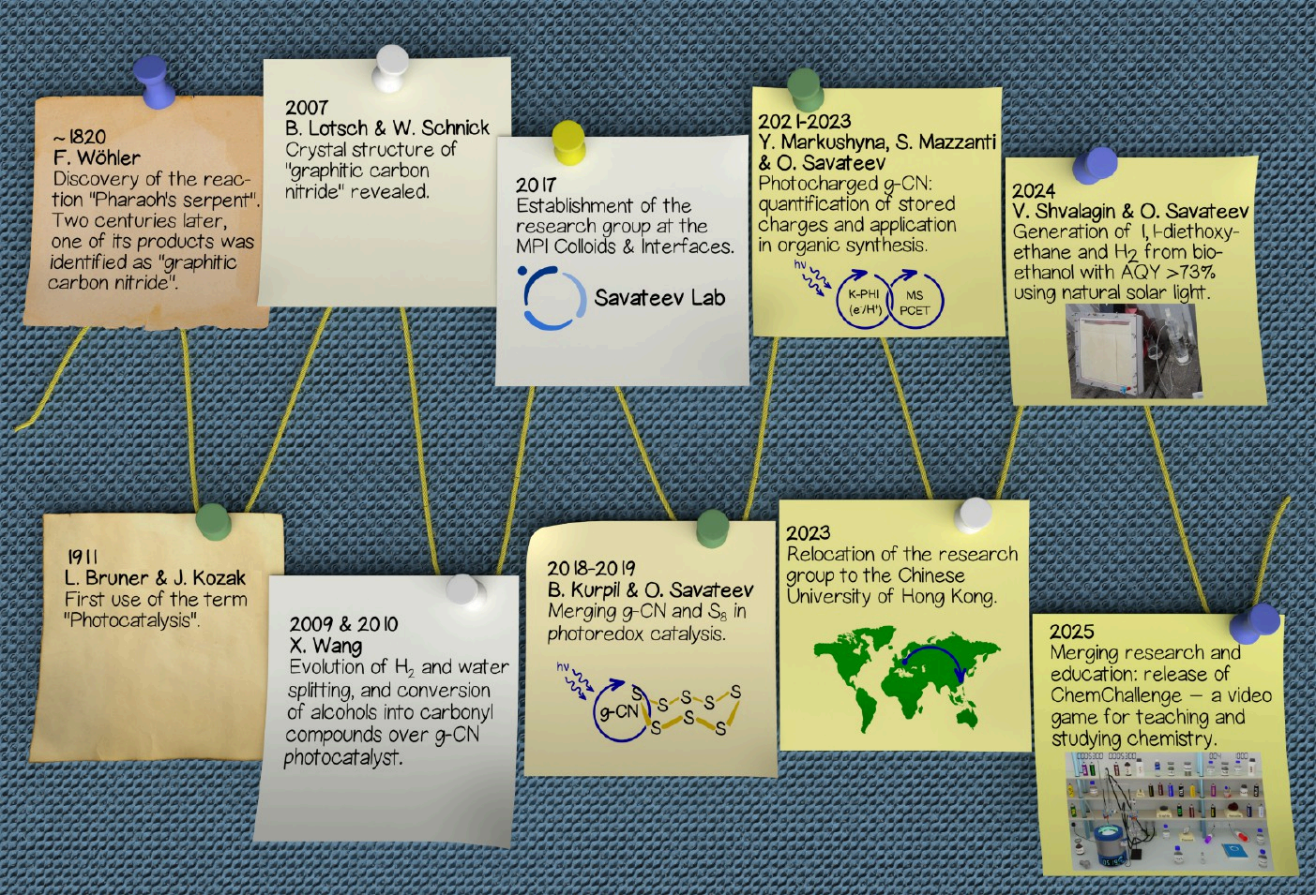
Historic snapshot of research in photocatalysis and carbon nitrides and the group’s contribution to “carbon nitride organic photocatalysis”. Photograph of the flat panel photoreactor is reproduced from ref 13. Available under a CC-BY 4.0 license. Copyright 2024 Shvalagin et al.^13^ Interface of the video game ChemChallenge is reproduced from ref 12. Available under a CC-BY 4.0 license. Copyright 2025 Savateev et al.^12^ Images of old and vintage paper sheets and a world map are licensed from Envato.com.

## Photophysics and Redox Properties of g-CNs

Depending on the chemical structure of their building units, g-CNs can be classified into two main categories: triazine-based and heptazine-based g-CNs.^14,15^ The structure and properties of these g-CNs have been extensively documented in reviews.^16^ Currently, the two most predominant structural variants of heptazine-based g-CNs employed in organic synthesis are melon-type g-CN and poly­(heptazine imides) (PHIs). Fragments of their chemical structures are shown in Figure 2. The melon-type g-CN, often referred to as polymeric carbon nitride (PCN), consists of linear chains of heptazine units covalently linked via bridging amino groups.^17^ In contrast, PHIs feature a two-dimensional network of heptazine units connected through imide linkages, with charge-compensating cations (M^+^ = K^+^, Na^+^, H^+^) residing between the layers.^18–20^ These structural variations critically influence the electronic structure, surface chemistry, and redox site accessibility, making g-CNs attractive scaffolds for photocatalytic and electrocatalytic transformations. Consequently, triazine-based g-CNs have been more frequently employed in photocatalytic water splitting than in organic synthesis.^21^ This narrow application scope stems from their challenging preparation and the wider bandgap that necessitates UV irradiation. The high energy of UV light often compromises the reaction selectivity, thereby hindering the general adoption of triazine-based g-CNs for selective organic transformations.

**Figure 2:**
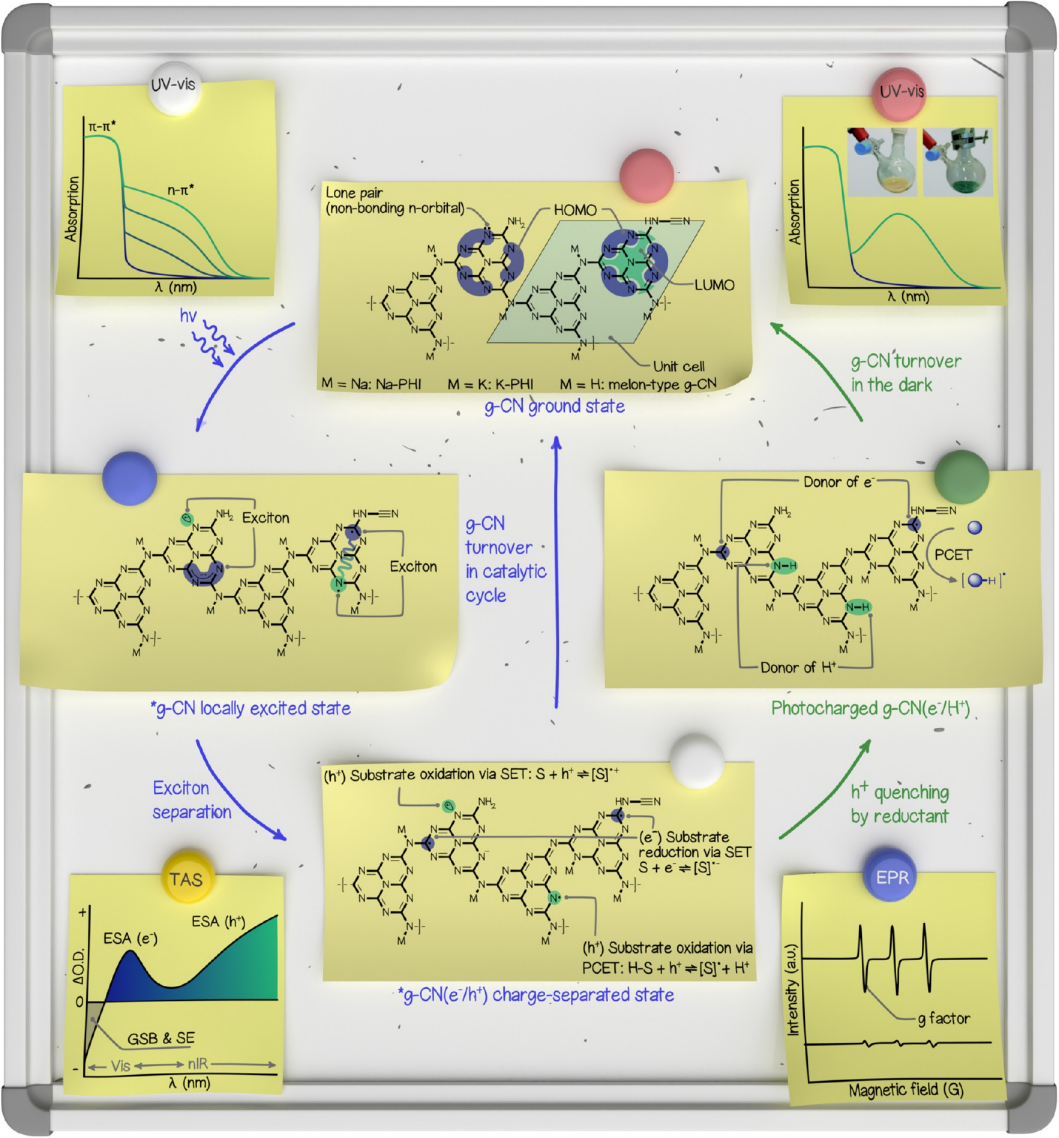
A summary of heptazine-based g-CN chemical structures, the structure of the g-CN electronically excited state, and schematic illustration of data obtained upon investigation of this class of materials through several spectroscopic techniques. Photographs of K-PHI slurry and photocharged K-PHI are reproduced from ref 22. Available under a CC-BY 3.0 license. Copyright 2019 Markushyna et al.^22^

Knowledge of the structure of redox sites in electronically excited g-CN and their potentials enables the rational design of chemical transformations of organic compounds using this class of sensitizers. Figure 2 collects these pieces of information. Electron transfer between the photocatalyst’s electronically excited state and substrates is governed by the laws of nature. Therefore, the same photocatalytic reaction may be enabled by many sensitizers, both homogeneous, such as organic dyes and transition metal complexes, and heterogeneous, including g-CNs.^23^ A combination of several features collectively allows g-CN photocatalysts to drive specific chemical reactions with higher efficacy and, in particular, higher quantum yield:^2^

- In UV–vis spectra of g-CNs, electronic transitions between the HOMO and LUMO (π–π*) are observed as, typical for semiconductors, steep onset of absorption at approximately 460 nm.^10^ However, if a g-CN possesses a substantial number of defects, which typically distort the conjugated system from being perfectly planar, additional absorption is registered in the visible range of the electromagnetic spectrum. This absorption band can correspond, for example, to electronic transitions between the nonbonding orbitals represented by nitrogen lone pairs and LUMO (n−π*), which is schematically shown in Figure 2.^1^ Excitation of g-CN featuring π–π* and n−π* transitions yields two kinds of redox sites: (1) strongly oxidative, represented by the photogenerated hole (h^+^) that is localized at the HOMO, and (2) mildly oxidative, represented by the radical cation localized at the nitrogen atom. Excitation of an electron from the n to π* orbital requires a photon of longer wavelength, such as 530–650 nm, while HOMO-to-LUMO transitions are enabled by 350–450 nm photons. Shining photons of a specific wavelength onto such a g-CN allows tuning the selectivity of a chemical reaction by modulating the oxidative power of the photocatalyst’s excited state. This idea is at the foundation of chromoselective catalysis.^24^
- Upon light absorption, a photon enables electron transfer from the HOMO to LUMO, affording g-CN in its locally excited state. The dynamic properties of the g-CN excited state are effectively probed and analyzed through advanced transient characterization techniques, which are well-established in the field of materials science and photophysics. In recent years, transient absorption spectroscopy (TAS) has emerged as a powerful and widely adopted experimental technique for investigating the ultrafast dynamics of photogenerated excitons, free electrons, and holes with high temporal resolution.^25^ The negative signal of TAS originates from two physical phenomena: ground-state bleach (GSB) and stimulated emission (SE). GSB arises from the photoexcitation of g-CN, which generates excitons that deplete the population of electrons in the ground state, thereby reducing absorption at ∼400–450 nm. On the other hand, SE refers to the radiative process resulting from the recombination of excitons, leading to the emission of photons at characteristic energies. The positive signal, excited-state absorption (ESA), in the visible range of ∼500–700 nm is attributed to light absorption by the species, which in Figure 2 are denoted as photogenerated electrons (e^–^), while that stretching from ∼800 nm into the nIR range is attributed to photogenerated holes (h^+^). The relative intensities, lifetimes, and kinetic evolution of the positive and negative signals in transient absorption spectra serve as indicators of the efficiency of exciton dissociation into free charge carriers. A pronounced and sustained positive signal of ESA suggests the formation of a long-lived charge-separated state, which is highly favorable for catalytic reactions. Conversely, excessively rapid recovery of the GSB signal may indicate a high rate of exciton recombination, highlighting the need to suppress such processes through strategies such as interface engineering or defect control. These observations offer valuable insights for the rational design of g-CN-based materials with enhanced photocatalytic performance.
- For an exciton to dissociate into a free electron (e^–^) and a hole (h^+^), which in Figure 2 are shown in the structure denoted as the *g-CN­(e^–^/h^+^) charge-separated state, it must overcome an energy barrier called the exciton binding energy (*E*_b_).^26^ The *E*_b_ of g-CN can be experimentally determined using temperature-dependent photoluminescence spectroscopy, with the reported values generally falling within the range of tens to over a hundred millielectronvolts.^27,28^ Taking n-type g-CN as an example, electrons serve as the majority charge carriers, resulting in the accumulation of positively charged holes upon excitation with light. This charge separation can be detected through steady-state surface photovoltage (SPV) spectra and Kelvin probe force microscopy (KPFM), which exhibit a positive response in the surface potential.^29^ The amplitude of this signal correlates positively with exciton dissociation efficiency.
- In the charge-separated state, g-CN functions as a versatile reactive species, capable of serving as both a one-electron oxidant and a reductant. A hole (h^+^ in Figure 2) can oxidize organic substrates via single-electron transfer (SET). The chemical structure of h^+^ is represented by a N-centered radical, which is also a strong hydrogen atom acceptor. Therefore, the g-CN electronically excited state can generate radicals from organic substrates via hydrogen atom transfer (HAT) or proton-coupled electron transfer (PCET) mechanisms. The intrinsic sp^2^-hybridized nitrogen-rich framework endows g-CN with a property of a weak base; the apparent p*K*_a_ of a conjugate acid, i.e., protonated melon-type g-CN, is approximately 6.6, whereas that of PHI exceeds 7.1.^2^ PHI is a stronger base compared to melon-type g-CN, apparently due to the presence of deprotonated imide linkers. Basic properties of g-CN make this class of materials similar to a “proton sponge”, which facilitates the polarization of C–H and ionization of O–H bonds in the substrate. Conversely, electrons (e^–^ in Figure 2) can participate in reductive processes through analogous electron transfer pathways. The radical intermediates generated upon substrate oxidation or reduction can be trapped by specific spin-trapping agents and characterized by electron paramagnetic resonance (EPR) spectroscopy, thereby serving as a powerful diagnostic tool for elucidating reaction mechanisms in photocatalytic and radical-mediated processes. Furthermore, the interaction between g-CN in its charge-separated state and substrate molecules can be probed by monitoring the degree of fluorescence quenching and shifts in binding energy as measured by X-ray photoelectron spectroscopy (XPS).^4^ These characterization techniques enable the elucidation of fundamental physicochemical properties of g-CN, thereby providing critical guidance for the design and optimization of organic synthesis reactions.

## Application of g-CNs as Photocatalysts in Organic Synthesis

Years of research and successful cooperation with experts in their fields, whose contributions are acknowledged below, now allow us to generalize and put our earlier findings in a broader context. Guided by the necessity to develop more atom-efficient methods to introduce sulfur into organic compounds, our team was the first to use elemental sulfur (S_8_) as the reactant (it is incorporated into the product) and the sacrificial electron acceptor in photocatalytic transformations mediated by g-CN. Compared to common sulfurating agents, such as Lawesson’s reagent, using S_8_ makes organic synthesis more atom-efficient. However, there is an even more fundamental reason linked to the higher chemoselectivity of reactions when S_8_ is employed instead of O_2_. In net-oxidative photocatalytic transformations, oxygen is typically used as a sacrificial oxidant. The formation of reactive oxygen species (ROS), such as the superoxide radical,^30^ prompted us to investigate sulfur as an alternative oxidant (Figure 3).

**Figure 3:**
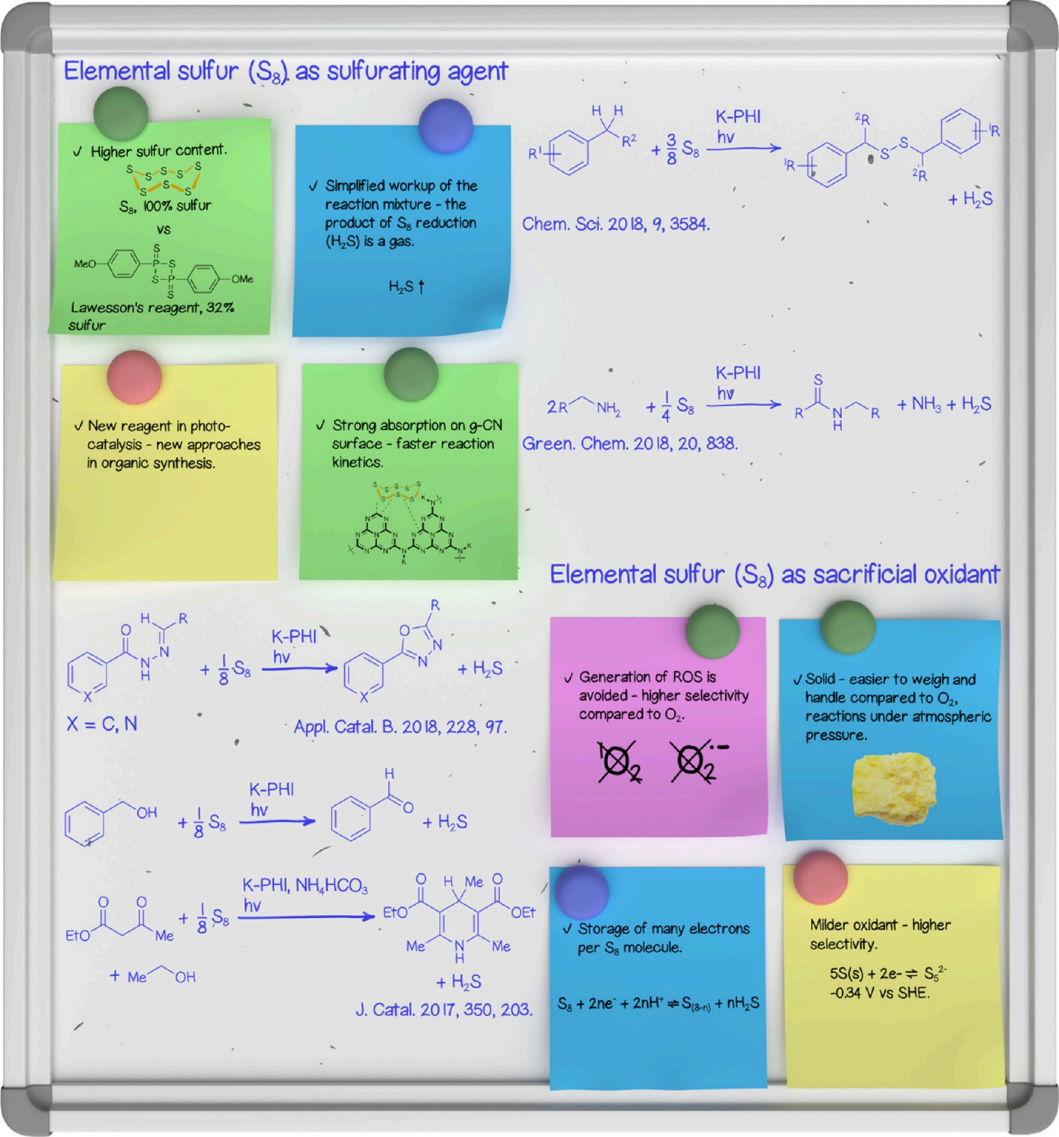
Photocatalytic transformations of elemental sulfur mediated by g-CN. A photograph of a chunk of sulfur is licensed from Envato.com.

The synergy between g-CN and elemental sulfur arises from the distinct downstream chemistry accessed when S_8_, instead of O_2_, serves as the terminal oxidant. The photocatalytic reduction of S_8_ by g-CN proceeds via a stepwise multielectron transfer process, generating polysulfide species and sulfur-centered radicals. These intermediates not only function as a reversible electron reservoir but also participate directly in C–S bond formation. This mechanism enables simultaneous photocharge storage and productive cross-coupling while effectively avoiding overoxidation of sensitive substrates. In particular, the yield of oxadiazoles-1,3,4 (Figure 3) upon the cyclization of acylhydrazones was lower when O_2_ was used as a sacrificial oxidant, compared to S_8_.^31^ The reason is the nucleophilic attack of the superoxide radical at the hydrazone C=N bond, followed by its cleavage. This side reaction does not proceed in the case of using S_8_, which results in higher selectivity and higher yield.

Under irradiation with blue light, a combination of K-PHI and S_8_ drives the selective oxidation of benzylic alcohols to the corresponding carbonyl compounds. In this example, the uncontrolled reactive oxygen chemistry associated with O_2_ and ROS and the overoxidation of benzaldehyde to benzoic acid, a common issue in photocatalysis under aerobic conditions, is effectively avoided. The in situ formed aldehydes can be intercepted in downstream condensations. For example, a Hantzsch-type assembly with β-ketoesters and ammonium bicarbonate furnishes highly substituted 1,4-dihydropyridine scaffolds.^32^

Until now, we have explored S_8_ as a sulfurating agent in combination with several classes of organic molecules. For example, a tentative HS_8_^•^ radical that is generated upon S_8_ reduction participates in a reaction with the ArCH_2_^•^ radical, which is also generated photocatalytically and delivers (ArCH_2_S)_2_.^33^ As a byproduct of the oxidative coupling of benzylamine, H_2_S can undergo further addition to the imine’s C=N bond, followed by dehydrogenation through a photocatalytic Kindler reaction mediated by K-PHI.^34^ Therefore, thioamides are synthesized from benzylic amines and S_8_. Notwithstanding the high selectivity of the reaction, the byproduct, H_2_S, is readily manageable.

Practically, S_8_ is a convenient solid reagent that avoids gas-handling and can improve operational simplicity and substrate tolerance, although issues of solubility, g-CN surface sulfuration, and possible H_2_S formation must be managed. Overall, the g-CN/S_8_ manifold furnishes an atom-economical and mechanistically controlled route to sulfur incorporation that complements, and in many cases outperforms, O_2_-driven photocatalysis in selectivity and catalyst longevity.

Figure 4 illustrates the versatility of g-CN photocatalysts in divergent organic synthesis using toluene as the starting compound. Depending on the reaction partner, O_2_ or S_8_, g-CN converts toluene or other alkylarenes into aromatic aldehyde or dibenzyldisulfide, respectively.^33,35^ Benzaldehyde and dibenzyldisulfide are the first-generation products of photocatalysis in this example. Using simple organic transformations, these first-generation products are converted into substituted imines, chalcones, oximes, acylhydrazone derivatives, or thiouronium salts. Depending on the reaction conditions, by employing g-CN photocatalysis, these five reactants serve as precursors to seven classes of more complex organic compounds: substituted ethanediamines,^3^ 1,3-disubstituted 5,6-dihydropyrrolo­[2,1-*a*]­isoquinoline (DHPIQ) derivatives,^36^ substituted γ,γ-dichloroketones,^37^ substituted cyclopentanoles,^38^ substituted oxadiazoles-1,2,4 ^1^ and oxadiazoles-1,3,4,^31^ and benzylic sulfonyl chlorides.^39^

**Figure 4:**
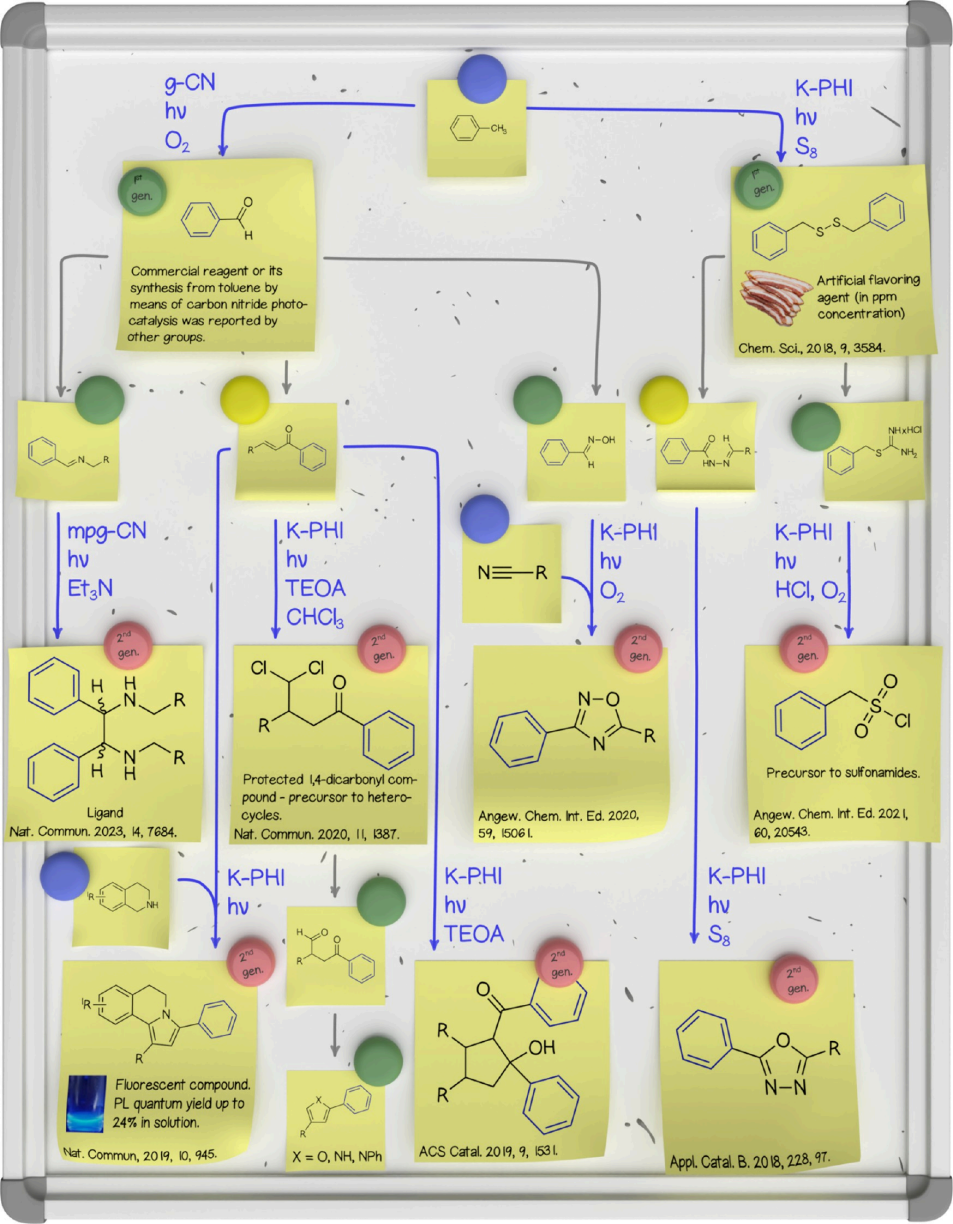
A summary of photocatalytic transformations developed by the group that start with toluene. A photograph of DHPIQ solution fluorescence is reproduced from ref 36. Available under a CC-BY 4.0 license. Copyright 2019 Kurpil et al.^36^ A photograph of bacon is licensed from Envato.com. PL, photoluminescence; TEOA, triethanolamine.

Synthesis of DHPIQ derivatives is an intriguing example illustrating that photocatalysis can alter the selectivity of a chemical reaction or activate a different reaction pathway. In the dark, the addition of tetrahydroisoquinoline to the β-carbon atom of chalcone takes place. This product of the aza-Michael reaction does not undergo cyclization into the DHPIQ derivative, even upon the addition of a photocatalyst and light irradiation. DHPIQ derivatives are formed only when the reagents K-PHI and light are combined in one pot.

The synthesis of first- and second-generation photocatalysis products was achieved without the addition of complex auxiliary reagents, using only transition metal-free g-CN photocatalysts and inexpensive reagents. We hope that the developed methods contribute to the toolbox for more sustainable methods in organic synthesis. Many of the compounds obtained using the developed photocatalytic methods possess interesting properties and have potential applications. For example, DHPIQ derivatives show a strong blue fluorescence in solution. Substituted γ,γ-dichloroketones serve as precursors to the synthesis of heterocycles, while benzyl sulfonyl chlorides serve as precursors to sulfonamides, constituting a large class of antibiotics. In parts per million concentration, dibenzyldisulfide is approved for usage as an artificial flavoring agent.

Until now, the group has been focusing on the application of g-CN as a sensitizer primarily without adding transition metals. However, in cooperation with Prof. Ivo F. Teixeira (Federal University of São Carlos, Brazil),^40^ Prof. Burkhard König (University of Regensburg, Germany),^41,42^ Prof. Gianvito Vilé (Politecnico di Milano, Italy),^43^ and Prof. Bartholomäus Pieber (Institute of Science and Technology Austria, Austria)^44^ as the leading partners in the projects, g-CNs were used as supports of transition metal single atoms or sensitizers in dual transition metal photocatalysis.

## Application of g-CNs as Energy Storage Materials and Reductants in Organic Synthesis

Conventional photocatalytic organic reactions are conducted under continuous illumination (blue path in Figure 2). As inferred from this general photoredox catalytic cycle, the g-CN photocatalyst enables the redox reactions while being constantly regenerated. To close the catalytic cycle, both the electron donor and the electron acceptor are required. However, when the reaction mixture is missing substrates that can act as oxidants, the electrons and charge-compensating ions, such as H^+^, are accumulated in g-CN. Such a reduced g-CN is commonly termed as “photocharged g-CN”. In a series of publications, we quantified the density of electrons stored in heptazine-based g-CN, in mol g^–1^ and using other units, by quenching the photocharged state with the redox indicator dimethylviologen dichloride (MV^2+^ × 2Cl^–^). This parameter depends critically on the structure of the carbon nitride material.^22^ The highest value for PHI reaches up to 1 mmol of reactive electrons per 1 g of the material. This corresponds to approximately 1 electron being stored over 4 heptazine units. On the other hand, the conditions under which photocharging is conducted also affect the density of stored electrons. Given the complex dependence, we established a database of photocharged materials, which collects many parameters of the photocharging process and the density of electrons stored in g-CNs and other materials.^45^ Although the precise mechanism underlying electron storage remains to be fully elucidated, the physicochemical characteristics exhibited by photocharged g-CN are largely consistent across a series of publications on this topic. The EPR spectrum of photocharged g-CN exhibits an obvious signal centered at approximately 3350 G, which is assigned to electrons trapped within the material.^46^ More intuitively, the photocharged g-CN typically exhibits emergent absorption features in the long-wavelength region (typically 500–800 nm), which are perceived as a blue, green, gray, or, in the case of using ammonium formate as a sacrificial electron donor, even black color of the material.^3^ The color depends on the structure of PHI and the density of stored electrons. While photocharged PHI­(e^–^/H^+^) is sensitive to oxygen, PHI­(e^–^/NH_4_^+^) is significantly less reactive; judging by the color transition from black to yellow, this photocharged state can survive in air for more than 20 min. Apparently, ammonium counterions exert a strong stabilizing effect on the stored electrons. In the context of the application of g-CN as a photocatalyst under continuous illumination, the change in the material color from yellow (before the start of the reaction) to green or blue (photocharged state, PHI­(e^–^/H^+^)) serves as a convenient indicator to mark the full consumption of the electron acceptor.^33^

It has been known that the electrons and protons stored in the photocharged g-CN­(e^–^/H^+^) can recombine and form H_2_ in the dark upon the addition of cocatalysts.^47^ Our group pioneered applying the photocharged g-CN as a reductant in organic synthesis (green path in Figure 2 and Figure 5).^48^ In particular, we used K-PHI­(e^–^/H^+^) to reduce a series of arylhalides to the corresponding aromatic hydrocarbons. The photocharged state is engaged in multielectron/proton transfer processes, including the 6e^–^/6H^+^ reduction of nitrobenzene to aniline.

**Figure 5:**
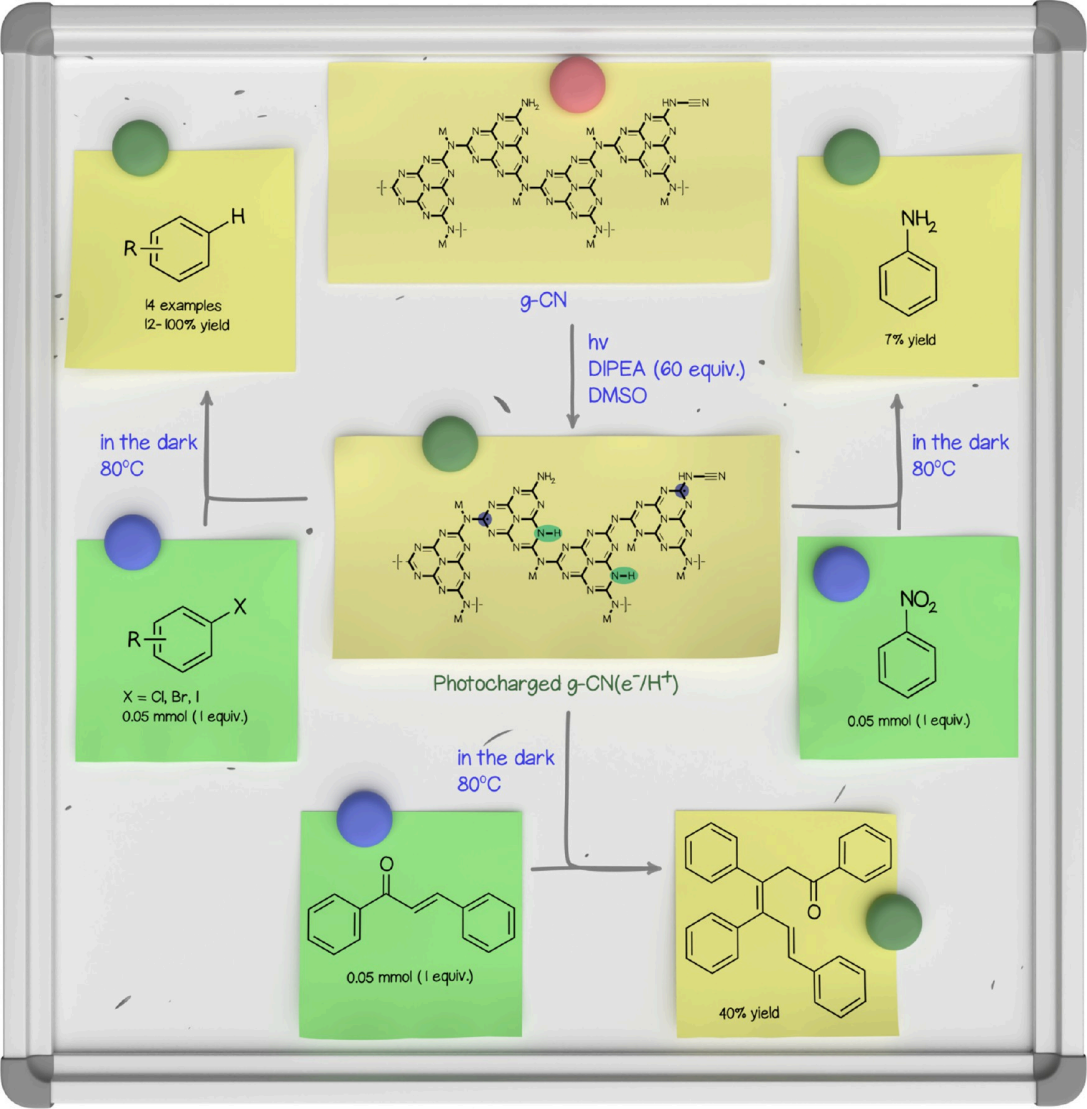
Application of photocharged g-CN­(e^–^/H^+^) as a reductant in the conversion of organic molecules. Quantities of reactants in a typical experiment: g-CN (80 mg), *N*,*N*-diiso­propyl­ethyl­amine (DIPEA, 3.2 mmol, 64 equiv), arylhalide/chalcone/​nitrobenzene (0.05 mmol, 1 equiv).

Unlike common reductants, such as LiAlH_4_ and NaBH_4_, which supply hydride anions, g-CN­(e^–^/H^+^) serves as a source of electron(s) and proton(s). As such, the reduction of a substrate proceeds via open-shell intermediate(s). This aspect can result in a different reaction selectivity compared to that obtained when using hydridic reductants. In addition, the density of electrons and charge-compensating ions stored in g-CN­(e^–^/H^+^) can also influence the reaction selectivity and reactivity of the photocharged state.^3,48^ For example, irradiation of a mixture of chalcone, K-PHI, and TEOA at 465 nm produces cyclopentanole derivatives (see Figure 4), while adding chalcone to the photocharged K-PHI, K-PHI­(e^–^/H^+^), and maintaining the mixture at 80 °C gives a derivative of hexadienone (Figure 5). The difference in the regioselectivity of chalcone transformation is likely to depend on the concentration of reductive species in the reaction mixture. Thus, upon continuous irradiation, the concentration of electronically excited K-PHI acting as a reductant at any point in time is significantly lower than the concentration of the substrate. As such, SET must be involved in the mechanism. On the other hand, K-PHI­(e^–^/H^+^), which could serve as a multielectron/multiproton reductant, is available in the reaction mixture in the quasi equimolar ratio vs the substrate.

Organic synthesis driven by the photocharged state of g-CN, while less explored than direct photocatalysis, represents a valuable strategy for reductive transformations. These include processes requiring electron and proton transfers and, notably, the conversion of photosensitive substrates, thereby effectively circumventing potential photodegradation. Moreover, this unique capability also enables a spatiotemporal separation of energy capture and use, functioning like a “solar battery” for organic synthesis.

## Current Challenges and Future Development Prospects of Carbon Nitride-Mediated Organic Synthesis

Although g-CN is considered as a promising multifunctional platform for organic synthesis, carbon nitride organic photocatalysis is still in a relatively immature stage. A decade of research has elucidated that an efficient g-CN photocatalyst for organic synthesis should ideally integrate the following key characteristics: efficient charge separation and migration, suitable redox potentials, strong visible-light absorption, and high stability and recyclability. To promote the sustainable development of this research direction and realize the full potential of g-CN in organic synthesis, it is imperative to devote more systematic and in-depth efforts. Specifically, the following core challenges need to be prioritized and addressed in future studies.

- The precise modulation of the g-CN crystal structure represents an ongoing yet critical challenge, as crystallinity exerts a profound influence on its optical absorption, charge carrier transport, and redox properties. Although PHI-based g-CN exhibits a two-dimensional layered architecture, heterogeneities in morphological characteristics, surface area, defect density, and chemical composition across synthetic batches result in limited reproducibility in catalytic behavior.
- Owing to the inherently heterogeneous nature of photocatalytic processes occurring on the surface of g-CN, the adsorption and subsequent activation of substrates become highly crucial for driving efficient transformations. Yet, the concurrent activation of multiple substrates is considerably challenging in systems employing g-CN as the sole photocatalyst, without incorporating additional catalytic species. As a result, carbon nitride photocatalysis has not yet been established as a robust strategy for the synthesis of complex organic molecules, and the simultaneous optimization of both reaction selectivity and catalytic efficiency continues to represent a fundamental research obstacle.
- The utilization of photocharged g-CN to drive organic synthesis in the dark has achieved encouraging progress, but the scope of applicable reactions remains largely confined to a limited set of reductive transformations, leaving the potential for diverse synthetic applications underexplored. Compared to direct photocatalysis, reactions mediated by photocharged g-CN may be limited by kinetic constraints in electron transfer, resulting in lower overall efficiency. Moreover, the finite electron storage capacity of the material inherently restricts the stoichiometric scale of reactions, thereby limiting practical substrate throughput.
- In situ characterization techniques have become indispensable tools for elucidating the mechanisms and active sites of carbon nitride-mediated organic reactions.^49^ Spectroscopic methods, such as in situ FTIR, Raman, EPR, and XPS, allow for real-time monitoring of reaction intermediates, surface interactions, and charge carrier dynamics. However, the inherent organic polymer structure of carbon nitride complicates the interpretation of spectral data, making it difficult to correlate signals with specific active sites. Many techniques also suffer from limited temporal or spatial resolution under realistic reaction conditions, particularly for capturing short-lived intermediates or surface-bound species at the solid–liquid interface. Key challenges include the need for techniques capable of selectively probing the catalyst surface at the molecular level under operando conditions, improving the time-resolution to capture ultrafast charge transfer and activation processes, and correlating spectroscopic signals with catalytic performance in a quantitative manner.
- Carbon nitride-mediated organic synthesis has achieved notable success at the laboratory scale, enabling a variety of organic transformations under mild and sustainable conditions. Nevertheless, its translation to industrial-scale applications remains constrained by numerous fundamental and technical barriers. A major limitation is the inefficient photon and mass transfer in large-scale photoreactors. Unlike batch-type small-scale experiments, industrial processes require continuous-flow systems that can ensure uniform light penetration and efficient mixing—conditions under which powdered g-CN tends to aggregate, settling out of reaction mixtures and reducing catalytic activity.^50^ Although preliminary studies have reported the immobilization of g-CN and its integration into continuous-flow systems, current implementations remain confined to gram-scale demonstrations.^51–54^ Furthermore, the energy consumption of artificial light sources needed to activate g-CN remains high, and solar-driven systems face issues related to weather instability and low energy density.

Overall, the advancement of carbon nitride-mediated organic synthesis hinges on the rational design of highly efficient, selective, and stable photocatalytic systems. Furthermore, the implementation of advanced characterization techniques enables mechanistic elucidation at the molecular level, while innovations in photoreactor engineering serve to broaden the scope and practicality of these photocatalytic reactions. The progress in this interdisciplinary field necessitates effective collaboration among organic chemists, materials scientists, and chemical engineers. In the context of big data development, artificial intelligence and machine learning are emerging as promising research frontiers for the development of novel photocatalysts. These computational approaches can significantly accelerate and optimize experimental processes, thereby reducing both temporal and material costs. Our research group has dedicated efforts to database development,^45,55^ with the aim of leveraging data-driven methodologies to provide robust support for carbon nitride-mediated organic synthesis.
